# Exploring the Effectiveness of Road Maintenance Interventions on IRI Value Using Crowdsourced Connected Vehicle Data

**DOI:** 10.3390/s25103091

**Published:** 2025-05-14

**Authors:** Riccardo Ceriani, Valeria Vignali, Davide Chiola, Margherita Pazzini, Matteo Pettinari, Claudio Lantieri

**Affiliations:** 1 Department of Civil, Environmental and Material (DICAM) Engineering, University of Bologna, 40136 Bologna, Italy; valeria.vignali@unibo.it (V.V.); margherita.pazzini2@unibo.it (M.P.); claudio.lantieri2@unibo.it (C.L.); 2 R&D and Innovation, Movyon S.p.A., 50013 Firenze, Italy; davide.chiola@movyon.com; 3 NIRA Dynamics AB, 58330 Linköping, Sweden; matteo.pettinari@niradynamics.se

**Keywords:** connected vehicles, international roughness index, road maintenance, pavement management system, transportation infrastructures

## Abstract

This work aims to investigate the effectiveness of road maintenance interventions by analyzing changes in the International Roughness Index (IRI) by means of crowdsourced connected vehicle data. For this purpose, 136 pavement maintenance interventions on a single lane were considered over a period between 2021 and 2024. A multiple linear regression model (R^2^ = 0.780) has been employed as statistical tool to assess the relationship between pre/post-intervention IRI scores and various factors. Despite the fact that results showed a general improvement in pavement condition, the effectiveness of the interventions was found to be influenced by several factors. In particular, intervention on the middle lane appears to be the most effective for enhancing section characteristics, and the effectiveness of maintenance on the overall condition of the section tends to be reduced as the number of lanes increases. Additionally, maintenance appears to be less effective in improving post-maintenance performance as the initial IRI value increases; this suggests that severely deteriorated sections may require more extensive rehabilitation strategies. The ultimate aim of study is to provide evidence to support the inclusion of crowdsource vehicle data in Pavement Management Systems (PMSs) to optimize maintenance planning and resource allocation.

## 1. Introduction

Road infrastructure is recognized to be an essential asset, comprising an intricate network of pavements that are deemed necessary for the safe and efficient movement of people and goods. The phenomenon under discussion has been shown to facilitate connections between businesses, industries, and consumers. Furthermore, it has been demonstrated that the phenomenon also promotes social equity, economic growth, and development [1,2]. It is also widely acknowledged that roadway infrastructure serves as the fundamental foundation of a nation’s economic activity and well-being [3]. However, over time, the integrity of road pavement surfaces is subject to deterioration due to the combined effects of traffic and climatic loading. This deterioration leads to continuously evolving ride-surface profiles that influence driver and passenger safety, ride comfort, stability, energy consumption, and vehicle maintenance costs [4]; it is therefore vital to ensure that these assets are durable and sustainable [5]. In order to address the aforementioned challenges, it is essential that road managers are equipped with advanced tools and technologies with which to monitor and assess changes across the pavement network [6]. Pavement quality monitoring techniques generally encompass two primary types of evaluation: functional inspections and structural inspections. Functional examinations are used to identify road surface irregularities, such as roughness, texture, and skid resistance. In contrast, structural inspections are used to assess the load-bearing capacity and integrity of the pavement under traffic loads. The outcomes from these evaluations are critical in formulating effective road rehabilitation and maintenance programs [7]. Such information is of pivotal importance in enhancing service quality and establishing the foundation of maintenance strategies. Three distinct types of road maintenance can be categorized: routine, preventive, and corrective; the main difference among them is the timing of the action in the pavement life span. Routine maintenance, frequently conducted on an annual basis, exerts a negligible influence on pavement performance. The objective of preventive maintenance is to improve functional conditions, whereas corrective maintenance involves major interventions once thresholds of deterioration are reached. A balanced approach to infrastructure maintenance necessitates the alignment of data collection efforts with the information essential for the accurate assessment of pavement conditions [8]. The rising costs of infrastructure maintenance underscore the necessity of implementing comprehensive management strategies that take into account the maintenance requirements of such facilities [5]. Future developments in this field are expected to encompass greater automation, seamless integration with other management systems, and enhanced accessibility through web-based platforms. These developments are expected to foster improved decision-making and infrastructure management. In this regard, the deployment of a fleet of vehicles equipped with in-vehicle sensors can offer road agencies a novel approach to the monitoring strategy of pavement networks [9,10,11,12].

### 1.1. Pavement Management System (PMS)

Pavement management systems (PMSs) are designed to optimize the maintenance and management of the road network, which ensures efficient use of resources and the sustainability of infrastructure. The American Association of State Highway and Transportation Officials (AASHTO) defines PMSs as “a set of tools or methods that assist decision-makers in finding optimum strategies for providing, evaluating, and maintaining pavements in a serviceable condition over a period of time” [13]. PMS software is characterized by its comprehensive functionality, encompassing modules for analytical processes, data collection, performance modeling, maintenance, and rehabilitation plans. A notable feature of PMS software is its capacity to facilitate reporting and visualization of functional dynamics at the project and network level [14,15,16]. The purpose of these systems is to assist maintenance management in the optimization of budget management and allocation in the context of deteriorated road assets [17,18,19]. Since their inception in the 1960s, PMSs have evolved to incorporate advanced technologies such as GIS, GPS, LiDAR, and predictive models into the process. This development has ensured comprehensive yet multi-linear planning [20,21]. Recent advances in data collection methodology have had a substantial impact on the accuracy of road condition assessments. This, in turn, has had a significant influence on the estimation of remaining service life and, consequently, the optimal choice of maintenance strategies [19].

### 1.2. International Roughness Index

The International Roughness Index (IRI) mathematically characterizes the longitudinal profile of pavements, originating from the World Bank’s 1982 Brazil experiment [22]. Recognized as a universally accepted metric, the IRI measures pavement smoothness by calculating the average longitudinal profile, capturing surface irregularities that induce vehicular vibrations [22]. In the Italian road network, ASTM E 1926-08 standard [23] defines the calculation of the IRI based on the longitudinal profile of elevations, utilizing the “Quarter Car” model. By quantifying road roughness, the IRI provides critical data in standardized units—[m/km] or [mm/m]. Transportation agencies have traditionally employed the IRI as a key threshold for road maintenance decisions [24]. Regular monitoring of IRI values enables these agencies to identify deteriorating roads, thereby optimizing resource allocation for maintenance and rehabilitation (M&R) activities. Table 1 delineates the IRI thresholds, categorizing pavement conditions as good, fair, or poor.

Regarding highways, Table 2 shows more strict thresholds to be applied. The limitations imposed by IRI on highways are stricter in comparison to other road types, due to the fact that such motorways are required to meet higher standards with regard to safety, comfort, and durability, in consideration of the higher operating speeds at which they are designed to function. More restrictive thresholds are imposed to ensure higher performance and service quality.

As technology advances, methods for collecting IRI data have become increasingly sophisticated and efficient, resulting in an abundance of data. This proliferation emphasizes the necessity for robust and accurate models to predict IRI values based on the collected data, ensuring effective pavement management and maintenance planning.

### 1.3. Road Roughness Calculation Using Connected Vehicles

The implementation of intelligent transportation systems (ITSs) to road networks can be enhanced by the integration of autonomous and connected vehicles. These innovative solutions offer a novel approach to the execution of IRI monitoring strategies, thereby facilitating the optimization of traffic management and safety. A plethora of studies have examined and corroborated the dependability of this particular data type in the context of road monitoring. Llopis-Castelló [27] analyzed the relationship between IRI values obtained through auscultation methods and those gathered by Connected and Autonomous Vehicles (CAVs), finding a moderate positive relation between them. Mahlberg [28] conducted a comparative analysis of the IRI values obtained by inertial profilers and crowdsourced connected vehicle data, utilizing a linear correlation model. The study yielded a significant linear correlation (n), with an R^2^ of 0.79 and a *p*-value of <0.001, thereby substantiating the reliability and validity of the method. In another study, Mahlberg [29] draws parallels between crowdsourced ride quality data and the performance of an industry-standard inertial profiler within a construction zone. The study examines the application of standard original equipment manufacturer on-board sensors from production vehicles to monitor ride quality before, during, and after construction activities, with a particular focus on observing improvements in the IRI. Du [30] presented a wireless, GPS-integrated system for measuring pavement roughness and calculating the IRI using acceleration data and power spectral density analysis, showing the system’s accuracy, with less than 10% error compared to laser methods. Using in-car vibrations, Liu [31] proposed a technique to calculate a large-scale pavement roughness measurement based on a semi-supervised learning method. The work carried out by Sandamal and Pasindu [32] defined an estimated IRI based on the peak and root mean square vibration collected by smartphones.

These findings indicate that connected vehicle roughness data serves as a reliable tool for network-level monitoring of pavement quality. These results suggest that connected vehicle roughness data are a viable tool for network-level monitoring of pavement quality.

The present paper aims to establish the foundations for an exploration of the potential of integrating pavement monitoring through connected vehicle data in highway environments. The study’s specific objective is to assess the capability to monitor the IRI around maintenance interventions. This will be achieved by evaluating how effectively the data capture improvements and whether this capability is influenced by factors such as the lane location of the intervention. The rationale behind this focus stems from the premise that maintenance interventions, by their very nature, are events that can be anticipated with a reasonable degree of certainty. This characteristic confers upon them a clear temporal reference point, thereby facilitating the association of changes in the data with specific intervals. To the authors’ best knowledge, this research is the first of its kind. The other studies previously mentioned on similar topics to those addressed in this article are more focused on the validation of roughness data generated by on-board vehicle sensors. They provide evidence of paramount importance in the research field. For this reason, it is believed that the presented contribution can fit into the current academic framework as a link between existing studies and applicability in the context of maintenance and forecasting strategies.

## 2. Methods

This study examines changes in the IRI on highway road sections undergoing maintenance interventions; only interventions on one lane have been taken into account for the purpose of the study. Multiple linear regression (MLR) has been selected as the statistical technique to explore the relationships between functional pavement performance and the differences in datasets and external variables. Linear regression models are often used to explore the relation between independent variables and continuous outcomes [33,34]. MLR models have been already widely used in the field of pavement performance prediction [35,36,37]. MLR is used to explain a portion of the observed variability through a set of regressors and their mutual interactions. The greater the explained variability, the more accurate the prediction [38]. MLR is based on the following (1):

(1) $$y_{i}=\beta_{0}+\sum_{j=1}^{p}x_{ij}\beta_{j}+\varepsilon_{i}$$

where *y_i_* is the dependent variable; *β*_0_ is the constant; *β_j_* is the coefficient associated with each regressor *x_ij_*, and *ε_i_* is the random error term.

Specifically, the analysis is based on daily values obtained from connected vehicles. An average IRI value was calculated and compared for the 60-day periods before and after each maintenance event. To minimize the influence of outliers, data corresponding to the 10 days immediately preceding and following the maintenance interventions were excluded from the calculations. The neglection of these 10-day spans is crucial because the data provided are only relative to the closure of the roadwork; no information about work zone opening is provided. The 10 days after the intervention are also neglected for two main reasons: first, to avoid reporting errors on the closing day; secondly, to allow for connected vehicle data stabilization.

### Data Description

Nira Dynamics AB provided data derived from a fleet of connected vehicles equipped with “original equipment manufacturer” OEM sensors. Nowadays, the company can rely on a total fleet of more than 2 million vehicles over Europe and North America which gather data on road pavement performances. Data gathering, aggregation, and anonymization are GDPR-compliant with current EU regulation. Information regarding the maintenance interventions was instead provided by Movyon S.p.A., the R&D department of the Italian highway authority. Connected vehicle data were recorded daily over the period from 21 September 2021 to 23 June 2024 and the corresponding maintenance data pertain to interventions carried out during this timeframe. In terms of spatial resolution, vehicle data were aggregated and anonymized by Nira Dynamics AB every 25 m across the full width of the road section; in the cases analyzed, 181 vehicles pass on average over the sections (max: 364, min: 47, sd: 74). The road network under analysis is located near the city of Bologna and comprises two road infrastructure types: highways and ring roads. The highway segment covers approximately 80 km, while the ring road extends about 25 km. Regarding the maintenance interventions, a total of 136 operations were selected for analysis over the same time span. These interventions were distributed randomly across the site to ensure representativeness. The study focuses exclusively on preventive and corrective maintenance events occurring on a single lane, spanning lengths greater than 25 m, and involving surface course interventions on flexible pavements. However, detailed information regarding the specific characteristics of the pavement surface layer was not provided. The fundamental parameters involved in the study are listed as follows in Table 3.

**Figure 1:**
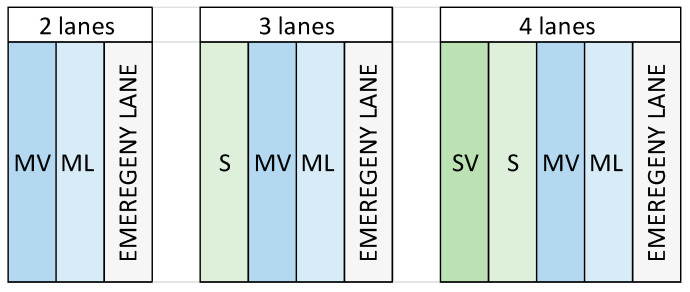
Lane identification code.

The total number of lanes involved in this study range from 2 to 4 (emergency lane excluded under the hypothesis that vehicles are forbidden to run over the lane according to Italian law); specifically, there were 15 cases of two-lane roads, 31 cases of three-lane roads, and ninety cases of four-lane roads. Of those lanes, the identification code of each one is shown in Figure 1 below.

In particular, 91 interventions have occurred on an ML lane; 29 on an MV lane; and 16 on an S lane, with 0 cases of interventions on an SV lane, where SV stands for “Sorpasso Veloce”, that is, the fast overtaking lane. IRImed_pre shows a medium value of 1.41 (min: 0.95, max: 2.79, SD 0.38), while IRImed_post is characterized by a medium value of 1.28 (min: 0.92, max: 2.49, SD 0.28). From those fundamental variables, arbitrary derived parameters have been calculated and are shown in Table 4. A logarithmic transformation was applied to parameters to mitigate the problem of possible non-linearity and pronounced asymmetry [39]. In addition, the log parameters have been multiplied by the total number of lanes in order to take into account the influence of the road section width.

## 3. Results

### 3.1. Normality of Data

Two main methods are used to assess normality: graphical and numerical (including statistical tests) [40]; the latter has the advantage of making an objective assessment of normality. This section presents findings of the normality tests using SPSS 27.0.1 software, conducted on the selected parameters: A and C. SPSS 27.0.1 refers to the Statistical Package for Social Science, which allows one to carry out a wide range of statistical analyses [41]. The normality of the data was assessed using the Kolmogorov–Smirnov test, because of the sample size (N = 136) [42]. The results of the normality tests for each variable are shown in Table 5 and reveal that most variables approximate normality.

### 3.2. Multiple Linear Regression

The outcomes of the multiple linear regression (MLR) analysis are reported in this paragraph. It investigates the relationships between the dependent variable C and the selected predictors: A, Lmtd/Ltot, and three categorical dummy variables (DUMMY_ML, DUMMY_MV, and DUMMY_S). The structure of the model dependent variable and predictor is reported in Table 6.

The application of a multiple linear regression model to this case study aims to evaluate the influence of the aforementioned variables on C, shedding light on potential patterns and their implications within the context of the study. The results of the model are reported in Table 7; they demonstrate robust explanatory power, with an R^2^ value of 0.780, indicating that approximately 78% of the variance in C is explained by the predictors. R^2^ achieves a high value only when the regression accurately predicts the majority of the ground truth elements within each group, taking their distribution into account [43]. Adjusted R^2^ is slightly lower at 0.773, which accounts for the number of predictors and confirms that the model is not overfitted; all variables seem to contribute to the model’s explanatory power. Furthermore, the Durbin–Watson (DW) statistic of 1.722 implies that the residuals are largely independent and that there is no severe autocorrelation. When there is a weak correlation between successive points, indicating a random distribution, the DW value approaches 2.0. For sample sizes where n > 100, the distribution can be considered random with 95% confidence if the DW value falls between 1.7 and 2.3 [44].

#### 3.2.1. ANOVA

The overall model is highly significant, as indicated by the ANOVA (Table 8) results (F = 115.788, *p* < 0.001). This finding confirms that the set of predictors collectively contributes to explaining the variability in C. The results indicate that the overall regression model is statistically significant in explaining the variance of the dependent variable C. The F-statistic value of 115.788, combined with a *p*-value of <0.001, confirms that the model provides a strong fit to the data. This suggests that the set of predictors collectively contributes to explaining the variability in C. The sum of squares attributable to the regression (5.182) accounts for the majority of the total sum of squares (6.647), highlighting the effectiveness of the model. The residual sum of squares (1.466) indicates that only a small portion of the variance remains unexplained. The mean square values (1.295 for regression and 0.011 for residuals) further support the substantial contribution of the predictors relative to the residual variance.

#### 3.2.2. Coefficients

The table of coefficients is shown in Table 9 and provides key insights into the role of each predictor. The coefficient value represents the magnitude of change in the predicted preference ranking. When standardized coefficients are used, interpretation is based on the standard deviations of the variables. Specifically, each coefficient indicates the number of standard deviations by which the predicted response changes in response to a one-standard-deviation change in a given predictor, assuming that all other predictors are held constant.

A emerges as the strongest predictor, characterized by a highly significant positive coefficient (B = 0.823, *p* < 0.001). The standardized beta value of 0.802 highlights its dominant role in explaining C variations; the positive relationship between A and C indicates that roads with poorer initial conditions, represented by higher values of A, are more likely. The positive relationship between A and C indicates that road sections have poorer initial conditions, represented by higher values of A. This suggests that roads that are in poorer condition prior to maintenance are less likely to show significant improvements following a single-lane intervention. Furthermore, both A and C are dependent on the total number of lanes, highlighting the amplifying effect of road complexity, as roads with more lanes are associated with higher A and higher C as a consequence. This finding suggests that addressing roughness on multi-lane roads presents additional challenges, likely due to the increased difficulty in achieving uniform improvements across all lanes. Furthermore, the positive coefficient of A suggests that higher initial roughness (ln(IRImed_pre)) contributes to residual roughness after maintenance (ln(IRImed_post)), reflecting the limited effectiveness of standard maintenance interventions on severely deteriorated roads. This finding indicates that conventional maintenance strategies may not be efficacious in cases of severe deterioration. In order to achieve the most favorable outcome, it is advisable to consider the implementation of measures in the section prior to the onset of degradation, or the execution of maintenance procedures that encompass both the functional and structural elements of the road package.

Lmtd/Ltot exhibit a significant but negative relationship with the dependent variable C (B = −0.853, *p* < 0.001). Its standardized beta coefficient of −0.306 suggests less impact compared to A. Since maintenance is applied on a single lane (Lmtd = 1), the ratio Lmtd/Ltot varies decrease as Ltot increases. The regression results indicate that as the total number of lanes increases, the C value decreases. This suggests that the impact of maintenance on the overall road condition, as measured by the roughness indicator C, becomes less pronounced on roads with a greater number of lanes.

The coefficient for DUMMY_MV (B = −0.086, *p* < 0.001) is also significant, though the effect size is smaller (Beta = −0.160). This dummy variable likely captures categorical effects, such as specific maintenance conditions or strategies, which negatively affect C. The negative sign of the coefficient implies that when maintenance is performed on the specific lane under evaluation (DUMMY_MV = 1), the value of C decreases compared to cases where maintenance is carried out on other lanes (DUMMY_MV = 0). This indicates that interventions directly targeting the lane being evaluated seem to be more effective in reducing the post-maintenance road roughness for the overall road section.

DUMMY_S (B = −0.065, *p* = 0.054) is at the limit of 95% significance; also, in this case, the effect is small (Beta = −0.094). This result tends to indicate that interventions carried out on the shoulder median lane seem to be effective in reducing the post-maintenance roughness but with a lower impact on the section with respect to the middle lane.

The variable DUMMY_ML does not show significant relationships with C (*p* > 0.05) and has been excluded by the software from predictors, so it can be defined as a non-significant predictor the for variable C. This result may be influenced by the fact that the MV lane experiences lower vehicular traffic (but a higher proportion of heavy vehicles), and, therefore, the lack of statistical significance may not be attributed to the inefficacy of the maintenance intervention, but rather to the reduced presence of vehicles in that lane, and consequently of data sources.

These considerations highlight that, although the effect of maintenance on a single lane appears to be diluted as the number of lanes increases, the effect also seems to vary depending on which lane the maintenance is performed on. Therefore, in the presence of monetary constraints—when maintenance can only be carried out on a single lane (assuming equal conditions across all lanes involved)—one may refer to the previously discussed findings in order to identify the intervention that yields the most favorable perceived outcome across the entire road section.

The diagnostic statistics confirm that the regression model is reliable; Variance Inflation Factors (VIFs) [45] for all predictors are below 1.6, indicating no multicollinearity issues [46]. This suggests that the predictors are sufficiently independent of one another, ensuring the model’s stability.

From the table of coefficients (Table 9), we can find Equation (2), which represents the *C* value.

(2) $$C=0.441+0.823A-0.853Lmtd/Ltot-0.086DUMMY_{MV}-0.065DUMMY_{S}+e$$

Given that *C* = ln(*IRImed_post*) × *Ltot*, we can express the value of *IRImed_post* for the maintained section as (3).

(3) $$IRImed\_post=e^{\left(\frac{1}{Ltot}*X\right)}$$

where *X* is given by (4)

(4) $$X=0.441+0.823A-0.853Lmtd/Ltot-0.086DUMMY_{MV}-0.065DUMMY_{S}+e$$

From the results of the regression, it might be said that performing maintenance on a single lane of a multi-lane road is less effective in improving the aggregate road condition compared to roads with fewer total lanes. By accounting for (5), it can be seen that for *Ltot* = 2 (n = 15), *delta_IRI* = 0.48 (sd = 0.33); for *Ltot* = 3 (n = 31), *delta_IRI* = 0.12 (sd = 0.12); for *Ltot* = 4 (n = 90), *delta_IRI* = 0.08 (sd = 0.06).

*delta_IRI_med* = *IRI_med_pre* − *IRI_med_post* (5)

#### 3.2.3. Residual Analysis

Residual analysis in Table 10 indicates no substantial deviations from homoscedasticity or normality, meaning that all the observations tend to have the same variance and residuals are normally distributed with zero mean [47], and this is evidenced by the distribution of residuals and their alignment with the model assumptions.

**Figure 2:**
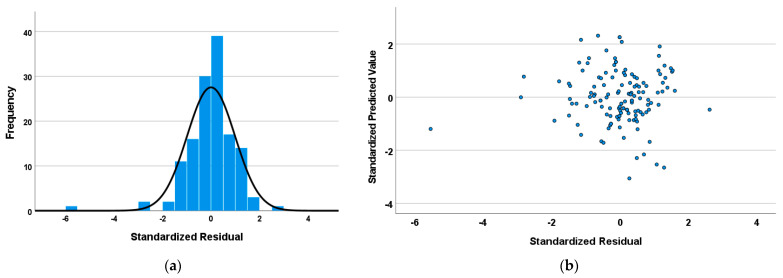
(**a**) Histogram; (**b**) scatter plot for standardized residuals.

As illustrated in Figure 2, the residual scatterplot is overlaid on the histogram of standardized residuals. The latter are plotted against the standardized predicted values; if the residuals are normally distributed, it ensures more reliable and interpretable *p*-values [48]. The absence of discernible patterns in the point distribution signifies that the model exhibits a high degree of fit [49].

## 4. Discussion and Conclusions

Supported by the findings from recent studies on the validity of data from connected vehicles in monitoring surface roughness [28,50], the objective of this study is to establish the foundations and propose the integration of such data into pavement management system (PMS) strategies for pavement maintenance and rehabilitation. The management of maintenance interventions on the road network is a matter of significant concern for road network administrators, primarily due to budgetary constraints and the management of the diverse interests of the stakeholders involved [51]. Given the aforementioned considerations, it is imperative that traffic engineers make choices that are consistent with the cost–benefit analysis. This will ensure that the optimal result is achieved among the various design solutions [52].

The presented study examines the effectiveness of single-line road maintenance interventions by looking at 136 cases and by analyzing the pre/post IRI using crowdsourced data from commercial connected vehicles. The interpretation of the statistical model (MLR) results applied to the dataset shows that the data provided by a fleet of connected vehicles can be a valuable and innovative tool, capable of improving the monitoring of road conditions and assessing the impact of maintenance interventions. These kinds of data have the potential to be capable of enhancing the monitoring of road conditions and assessing the impact of maintenance interventions. Analysis of the data confirmed that the maintenance intervention resulted in a reduction in the IRI, indicating an improvement in surface pavement quality. However, the effectiveness of the intervention was influenced by several factors, including the location of the maintained carriageway, its total number of lanes, and the initial road section condition. In addition, sections with a more pronounced initial IRI tended to show limited improvements to surface regularity, suggesting that interventions on severely deteriorated sections may require stronger and wider maintenance strategies. Furthermore, multiple linear regression showed how maintenance on different lanes can have different effects on the overall quality of the road section. The findings of this study indicate several practical implications for road managers. The utilization of crowdsourced data for pavement monitoring facilitates optimized intervention scheduling, enhancing resource allocation and diminishing maintenance costs; this approach facilitates the identification of sections requiring priority interventions, ensuring targeted and timely execution. Moreover, the variability in intervention effectiveness depending on the number of lanes and roadway location suggests the need for differentiated maintenance strategies. For instance, interventions on multi-lane roads should be designed with the consideration of the overall impact on road surface quality to ensure maximum benefits for the entire infrastructure.

In the future, the integration of these data with machine learning and predictive models could further improve the accuracy of the analyses [53], allowing the integration and implementation of reactive maintenance strategies with the aim of resource management optimization.

The quality of road pavements is monitored by the managing bodies, albeit only on one lane and most often on the slow lane. In the Italian motorway context, moreover, this type of activity is conducted biannually. It is imperative to contemplate the prospective benefits of data from connected vehicles in optimizing conventional monitoring methodologies. This is due to the fact that these vehicles travel on all available lanes, which can provide enhanced temporal and spatial resolution.

The utilization of this particular type of data has the potential to influence predictive maintenance, as well as reactive maintenance. The implementation of daily monitoring procedures for specific road sections has the potential to facilitate the identification of localized degradation. This, in turn, could enable maintenance personnel to execute targeted interventions.

Furthermore, authors suggest that future studies could investigate the influence of additional variables present in the literature, like weather conditions [54,55], traffic volumes, and pavement aging [56], to better understand the dynamics of pavement deterioration. In conclusion, this work provides a solid foundation for the use of crowdsourced data from connected vehicles in road maintenance management, contributing to more efficient and sustainable road infrastructure management.

## Figures and Tables

**Table 1:** IRI thresholds [25].

Condition	IRI (m/km)
Good	<1.5
Fair	1.5–2.7
Poor	>2.7

**Table 2:** IRI thresholds in highways (adapted from [26]).

Condition	IRI (m/km)
Good	<1.5
Fair	1.5–1.9
Poor	>1.9

**Table 3:** Fundamental parameters.

Fundamental Parameters	Unit of Measure (UoM)	Parameter Description
Ltot	n	Total number of lanes in the maintained section
DUMMY_ML	floating 0–1	If maintenance occurred in the shoulder lane (Marcia Lenta)
DUMMY_MV	floating 0–1	If maintenance occurred in the middle lane (Marcia Veloce)
DUMMY_S	floating 0–1	If maintenance occurred in the shoulder median lane (Sorpasso)
IRImed_pre	m/km or mm/m	Average daily IRI value for each maintained section 50 days before the maintenance event
IRImed_post	m/km or mm/m	Average daily IRI value for each maintained section 50 days after the maintenance event

**Table 4:** Derived parameters.

Derived Parameters	Parameter Description
Lmtd/Ltot	Ratio between the total number of lanes maintained (Lmtd) and the total number of lanes (Ltot)
A	Product between ln(IRImed_pre) and n_lines_tot
C	Product between ln(IRImed_post) and n_lines_tot

**Table 5:** Kolmogorov–Smirnov normality test results.

Kolmogorov–Smirnov ^a^			
	Statistic	Dof	Sign.
A	0.071	136	0.091
C	0.064	136	0.200 *

* This is a lower bound of effective significance. ^a^. Lilliefors correction.

**Table 6:** Dependent variable and predictors of the model.

Role	Variable	Description
Dependent variable	C	Product between ln(IRImed_post) and n_lines_tot
Predictors	A	Product between ln(IRImed_pre) and n_lines_tot
	Lmtd/Ltot	Ratio between the total number of lanes maintained and the total number of lanes
	DUMMY_ML	If maintenance occurred in the shoulder lane (Marcia Lenta)
	DUMMY_MV	If maintenance occurred in the middle lane (Marcia Veloce)
	DUMMY_S	If maintenance occurred in the shoulder median lane (Sorpasso)

**Table 7:** Multiple linear regression model results.

Model Recap ^b^				
R	R^2^	R^2^ Adjusted	Std. Error	Durbin-Watson
0.883 ^a^	0.780	0.773	0.106	1.722

^a^. Predictors: Lmtd/Ltot, A, ln_L, DUMMY_MV, DUMMY_S. ^b^. Dependent variable: C.

**Table 8:** ANOVA results.

ANOVA				
	Sum of Squares	Mean Square	F	Sign.
Regression	5.182	1.295	115.788	<0.001
Residual	1.466	0.011		
Total	6.647			

**Table 9:** Table of coefficients.

	Non-Standardized Coefficients		Standardized Coefficients			95% Confidence Interval for B		Collinearity	
	B	Std. Error	Beta	t	Sign.	Lower Bound	Upper Bound	Tolerance	VIF
Constant	0.441	0.083		5.281	<0.001	0.276	0.606		
A = ln(IRImed_pre) × Ltot	0.823	0.043	0.802	19.281	<0.001	0.738	0.907	0.972	1.029
Lmtd/Ltot	−0.853	0.136	−0.306	−6.258	<0.001	−1.122	−0.583	0.702	1.425
DUMMY_MV	−0.086	0.023	−0.160	−3.737	<0.001	−0.132	−0.041	0.921	1086
DUMMY_S	−0.065	0.033	−0.094	−1.945	0.054	−0.131	−0.001	0.716	1.139

**Table 10:** Residual statistics.

	Minimum	Maximum	Average	Standard Deviation
Predicted value	0.942	1.997	1.542	0.195
Residual	−0.587	0.276	0.000	0.105
Predicted value std.	−3.051	2.314	0.000	1.000
Standard residual	−5.491	2.581	0.000	0.977

## Data Availability

Restrictions apply to the datasets.

## Abbreviations

The following abbreviations are used in this manuscript:

IRI	International Roughness Index
MLR	Multiple Linear Relation
PMS	Pavement Management System
ML	Shoulder lane (Marcia Lenta)
MV	Middle lane (Marcia Veloce)
S	Shoulder median lane (Sorpasso)
SV	Fast overtaking lane (Sorpasso veloce)
